# The beneficial role of Asian-based RecurIndex test in the prognostic prediction in Chinese male breast cancer patients

**DOI:** 10.1038/s41598-021-87267-y

**Published:** 2021-04-07

**Authors:** Shuo Zhang, Beichen Liu, Mengli Zhou, Jintian Wang, Jinzhao Liu, Li Wang, Chao Yang, Yueping Liu, Shuyao Niu, Furong Du, Xiaohua Du, Ning Wang, Jiyu Tang, Chao Song, Yunjiang Liu

**Affiliations:** 1 Breast Center, The Fourth Hospital of Hebei Medical University, No.12 Jiankang Road, Shijiazhuang, 050011 Hebei China; 2 Department of Haematology, The Fourth Hospital of Hebei Medical University, No.12 Jiankang Road, Shijiazhuang, 050011 Hebei China; 3 The State Key Laboratory of Translational Medicine and Innovative Drug Development, Jiangsu Simcere Diagnostics Co., Ltd., No.699-18 Xuanwu Avenue, Nanjing, 210042 Jiangsu China; 4 Department of Breast Surgery, Xingtai People’s Hospital, No.16 Hongxing Street, Xingtai, 054031 Hebei China; 5 Department of Pathology, The Fourth Hospital of Hebei Medical University, No.12 Jiankang Road, Shijiazhuang, 050011 Hebei China

**Keywords:** Breast cancer, Cancer models

## Abstract

RecurIndex, a multigene profiling assay, can predict the risk of local recurrence and distant metastasis in female breast cancer (FBC), but its role in male breast cancer (MBC) remains unclear. In this study, the clinicopathological data of 43 consecutive MBC patients undergoing surgeries between 2009 and 2018 were retrospectively analysed. Their paraffin-embedded tissue sections were examined by RecurIndex test which comprised 2 models: recurrence index for local recurrence (RI-LR) and recurrence index for distant recurrence (RI-DR). Of 43 patients, there were 26 low-risk and 17 high-risk patients assessed by RI-LR, while 17 low-risk and 26 high-risk patients by RI-DR. For RI-LR, tumor N stage showed statistically significant (*P* < 0.001) between low- and high-risk patients; for RI-DR, differences were pronounced in tumor grade (*P* = 0.033), T stage (*P* = 0.043) and N stage (*P* = 0.003). In terms of clinical outcomes, the overall survival (OS) of low- and high-risk patients stratified by RI-LR showed no statistically significant differences (*P* = 0.460), while high-risk patients identified by RI-DR had a significantly worse distant recurrence-free survival (DRFS) (*P* = 0.035), progression-free survival (PFS) (*P* = 0.019) and OS (*P* = 0.044) than low-risk patients. Overall, RI-DR can effectively predict the DRFS, PFS and OS of MBC patients and identify those at low risk of recurrence, which may serve as a potential prognostic tool for MBC.

## Introduction

Male breast cancer (MBC) is a rare malignancy, approximately accounting for 1% of cancers in men and 1% of all breast cancers worldwide^1–3^. The incidence of MBC varies by ethnicities and geographical areas, with a high proportion of cases in Africa.^4^ According to the statistics, less than 0.2% of cancer-related deaths in men are ascribed to MBC^1,5^. In China, 0.4% newly diagnosed cases of MBC and 0.1% deaths were estimated in 2015^6^. Because MBC occurs at a very low incidence, its epidemiology, tumour behaviour, treatment options and prognosis are still unclear. Although MBC is similar to female breast cancer (FBC) in many ways^7^, the tumour biology is different. In contrast to FBC, MBC is usually diagnosed at an older age, with more frequent lymph node metastases and higher rates of estrogen receptor (ER)-positive tumours^7,8^, and it is more likely to occur within the setting of BRCA2 mutations rather than BRCA1 mutations^9^. In addition, a low androgen state is also considered as a known risk factor for MBC^10^.

Currently, the treatment strategies for MBC are mainly based on the data extrapolated from the females. Relatively little is known about the vital clinical and genomic differences between males and females. Previous studies showed that MBC patients had significantly shorter overall survival (OS) than the stage- and subtype-matched FBC patients^11,12^. Somatic genetic alterations typically occurring in ER-positive, human epidermal growth factor receptor 2 (HER2)-negative FBC are less common in MBC, such as TP53 and PIK3CA mutations and 16q loss^13^. Therefore, understanding the tumour biology and underlying mechanisms of sex-specific differences may contribute to optimizing the management of MBC^14^.

Multigene profiling assays, such as Oncotype Dx, Mammaprint and EndoPredict, have been developed for prognostic assessment of breast cancer based on European and American populations. Whether these multigene tests are appropriate to Asian populations needs further validation^15,16^. RecurIndex is a multigene prognostic test developed based on Chinese genes and clinicopathological features such as age, lymphovascular invasion (LVI), ER and lymph node status. With 44 scores as the threshold value, the accuracy of RecurIndex for predicting local–regional recurrence (LRR) in FBC can be up to 93%. Regardless of cancer subtypes or nodal status, RecurIndex can independently predict the risk of LRR in FBC^17^. Additionally, it is also demonstrated to predict the risk of distant recurrence (DR)^18^. In a recent study, 2 clinical-genomic models (recurrence index for local recurrence, RI-LR; recurrence index for distant recurrence, RI-DR) derived from RecurIndex test have been established, among which RI-DR is found more appropriate to Chinese FBC than Oncotype Dx in the prediction of DR^19,20^. To date, there are no studies reporting the application of RecurIndex in MBC. In this study, we first attempted to investigate the role of RecurIndex in predicting the prognosis of Chinese MBC patients.

## Results

### Clinicopathological features of included patients

Between 2009 and 2018, there were a total of 54 consecutive MBC patients who underwent surgeries in The Fourth Hospital of Hebei Medical University. After 5 cases of loss to follow-up and 6 unqualified tumour samples were excluded, 43 patients were finally enrolled into the study, with the median follow-up duration of 36 months. The clinicopathological features of patients are listed in Table 1. As it shown, the patients were at the age of (63.8 ± 12.5) years, 86.0% (37/43) were likely to have tumour grade of I-II, 97.7% (42/43) were ER/progesterone receptor (ER/PR)-positive and 88.4% (38/43) were HER2-negative. Only 30.2% of patients (13/43) had prominent LVI. Of these patients, the proportions of patients receiving adjuvant endocrine therapy, adjuvant chemotherapy and adjuvant radiotherapy were 67.4% (29/43), 48.8% (21/43) and 30.2% (13/43), respectively. According to the assessment of RI-LR, there were 26 (60.5%) low-risk and 17 (39.5%) high-risk patients, while based on RI-DR there were 17 (39.5%) low-risk and 26 (60.5%) high-risk patients.

**Table 1:** Clinicopathological features of included patients, n (%).

Characteristics	Description
Age, years, ($\stackrel{-}{x}$±s)	63.8 ± 12.5
**Lymphovascular invasion**	
Absent/focal	30 (69.8)
Prominent	13 (30.2)
**Tumor grade**	
I–II	37 (86.0)
III	6 (14.0)
**Tumor T stage**	
T1	19 (44.2)
T2	23 (53.5)
T3	1 (2.3)
**Tumor N stage**	
N0	23 (53.5)
N1	12 (27.9)
N2	6 (14.0)
N3	2 (4.7)
**Ki-67 expression**	
< 15%	5 (11.6)
15–30%	23 (53.5)
> 30%	15 (34.9)
**Estrogen receptor/progesterone receptor status**	
Positive	42 (97.7)
Negative	1 (2.3)
**Human epidermal growth factor receptor 2 status**	
Positive	5 (11.6)
Negative	38 (88.4)
**Adjuvant endocrine therapy**	
Yes	29 (67.4)
No	14 (32.6)
**Adjuvant chemotherapy**	
Yes	21 (48.8)
No	22 (51.2)
**Adjuvant radiotherapy**	
Yes	13 (30.2)
No	30 (69.8)
**Recurrence index for local recurrence**	
Low-risk	26 (60.5)
High-risk	17 (39.5)
**Recurrence index for distant recurrence**	
Low-risk	17 (39.5)
High-risk	26 (60.5)

### Association of RI-LR and RI-DR with clinicopathological features

The clinicopathological features of low- and high-risk patients stratified by RI-LR and RI-DR were compared in Tables 2 and 3, respectively. For RI-LR, tumour N stage showed statistically significant between low- and high-risk patients (*P* < 0.001; Table 2). For RI-DR, significant differences were presented in tumour grade (*P* = 0.033), T stage (*P* = 0.043) and N stage (*P* = 0.003) between low- and high-risk patients (Table 3).

**Table 2:** Correlation between RI-LR and clinicopathological features, n (%).

Characteristics	Low-risk group (*N* = 17)	High-risk group (*N* = 26)	*P*
Age, years, ($\stackrel{-}{x}$±s)	60.53 ± 13.87	65.88 ± 11.27	0.194
**Tumor grade**			0.055
I–II	25 (96.2)	12 (70.6)	
III	1 (3.8)	5 (29.4)	
**Ki-67 expression**			0.369
< 15%	16 (61.5)	7 (41.2)	
15–30%	7 (26.9)	8 (47.1)	
> 30%	3 (11.5)	2 (11.8)	
**Tumour T stage**			0.650
T1	12 (46.2)	7 (41.2)	
T2	13 (50.0)	10 (58.8)	
T3	1 (3.8)	0 (0.0)	
**Tumour N stage, n (%)**			< 0.001
N0	21 (80.8)	2 (11.8)	
N1	3 (11.5)	9 (52.9)	
N2	2 (7.7)	4 (23.5)	
N3	0 (0.0)	2 (11.8)	
**LVI**			0.356
Absent/focal	20 (76.9)	10 (58.8)	
Prominent	6 (23.1)	7 (41.2)	
**ER/PR status**			0.828
Positive	26 (100.0)	16 (94.1)	
Negative	0 (0.0)	1 (5.9)	
**HER2 status**			0.643
Positive	23 (88.5)	15 (88.2)	
Negative	3 (11.5)	2 (11.8)	

*LVI* lymphovascular invasion, *ER/PR* estrogen receptor/progesterone receptor, *HER2* human epidermal growth factor receptor 2, *RI-LR* recurrence index for local recurrence.

**Table 3:** Correlation between RI-DR and clinicopathological features, n (%).

Characteristics	Low-risk group (*N* = 17)	High-risk group (*N* = 26)	*P*
Age, years, ($\stackrel{-}{x}$±s)	64.45 ± 11.81	64.16 ± 13.20	0.942
**Tumor grade**			0.033
I–II	17 (100.0)	20 (76.9)	
III	0 (0.0)	6 (23.1)	
**Ki-67 expression**			0.140
< 15%	2 (11.8)	3 (11.5)	
15–30%	12 (70.6)	11 (42.3)	
> 30%	3 (17.6)	12 (46.2)	
**Tumour T stage**			0.043
T1	11 (64.7)	7 (26.9)	
T2	6 (35.3)	18 (69.2)	
T3	0 (0.0)	1 (3.8)	
**Tumour N stage, n (%)**			0.003
N0	15 (88.2)	8 (30.8)	
N1	1 (5.9)	11 (42.3)	
N2	1 (5.9)	5 (19.2)	
N3	0 (0.0)	2 (7.7)	
**LVI**			0.266
Absent/focal	14 (82.3)	0 (0.0)	
Prominent	3 (17.6)	10 (38.5)	
**ER/PR status**			1.000
Positive	17 (100.0)	25 (96.2)	
Negative	0 (0.0)	1 (3.8)	
**HER2 status**			0.643
Positive	1 (5.9)	4 (15.4)	
Negative	16 (94.1)	22 (84.6)	

*LVI* lymphovascular invasion, *ER/PR* estrogen receptor/progesterone receptor, *HER2* human epidermal growth factor receptor 2, *RI-DR* recurrence index for distant recurrence.

### Association of RI-LR and RI-DR with clinical outcomes

**Figure 1:**
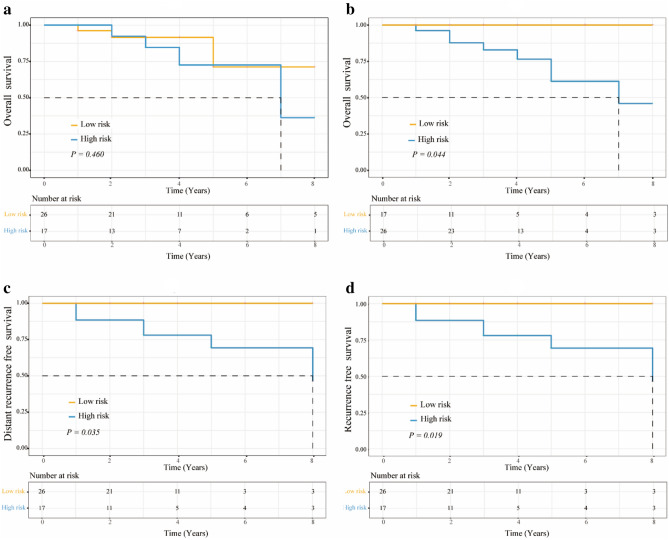
Comparison on the survival curves of low- and high-risk patients stratified by RI-LR and RI-DR. (**a**) Overall survival of low- and high-risk patients identified by RI-LR; (**b**) Overall survival of low- and high-risk patients identified by RI-DR; (**c**) Distant recurrence-free survival of low- and high-risk patients identified by RI-DR; (**d**) Progression-free survival of low- and high-risk patients identified by RI-DR.

For RI-LR, analysis of overall survival (OS) of low- and high-risk patients showed no statistically significant differences (*P* = 0.460; Fig. 1A). For RI-DR, however, high-risk patients had a significantly worse OS (69.2% *vs*. 100.0%, *P* = 0.044; Fig. 1B), distant recurrence-free survival (DRFS) (73.1% *vs*. 100.0%, *P* = 0.035; Fig. 1C), and progression-free survival (PFS) (65.4% *vs*. 100.0%, *P* = 0.019; Fig. 1D) than low-risk patients.

**Figure 2:**
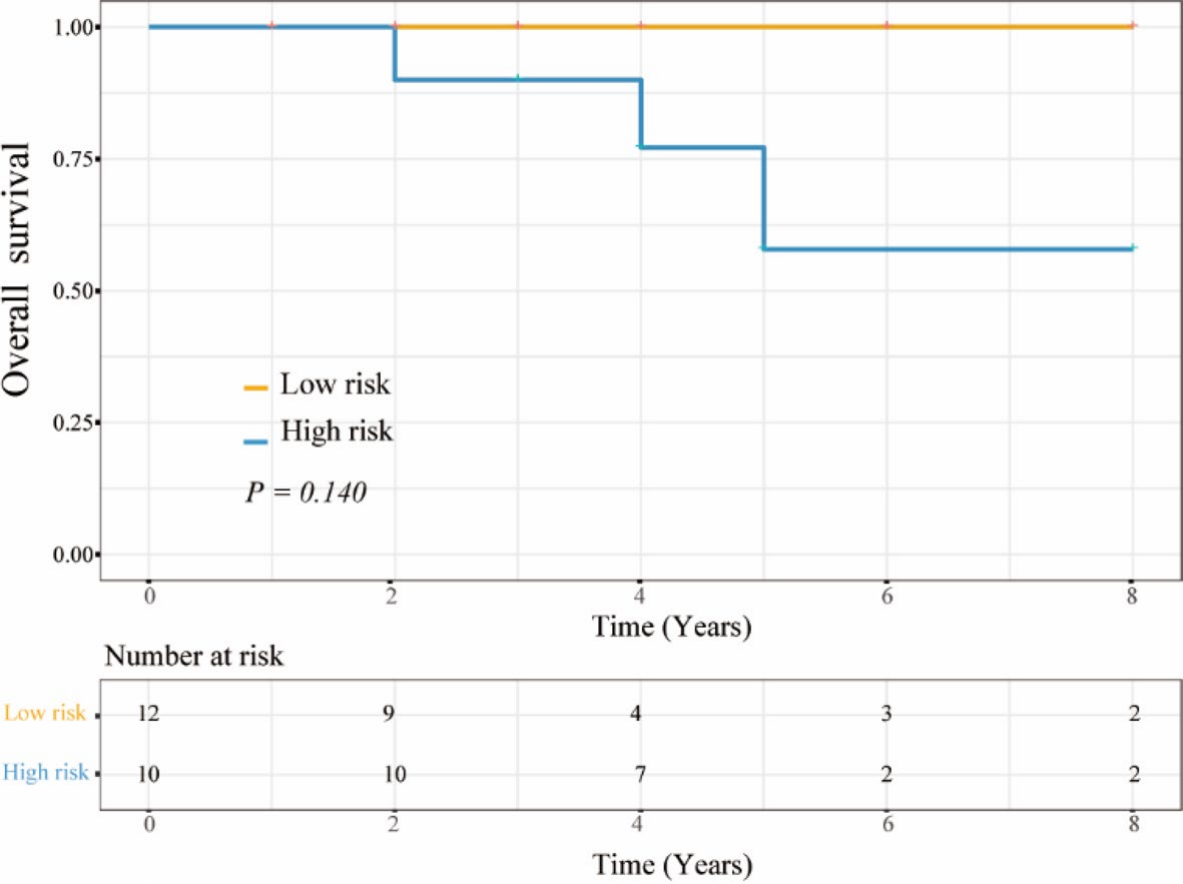
Comparison on the survival curves of low- and high-risk patients without adjuvant chemotherapy based on RI-DR.

During the follow up, 17 (100.0%, 17/17) low-risk patients identified by RI-DR did not suffer from any recurrences or deaths, while 9 (34.6%, 9/26) high-risk patients were subjected to recurrences or deaths. According to the subtypes, 22 patients not receiving adjuvant chemotherapy were screened out. Despite no statistically significant difference, the low-risk patients without adjuvant chemotherapy seemed to have a longer OS than the high-risk patients without adjuvant chemotherapy (*P* = 0.140; Fig. 2). Among the patients without adjuvant chemotherapy, 12 cases were predicted at low risk of recurrence. The final follow-up showed that these 12 patients did not experience any recurrences or deaths. All these findings suggested that RI-DR was conductive to identifying the patients at low risk of recurrence for MBC, and these patients might be exempt from postoperative adjuvant chemotherapy.

## Discussion

Genomic testing is thought to play an important role in aiding clinical decision-making and better balancing the efficacy and detriments of adjuvant therapies, which is valuable in avoiding overtreatments or harmful treatments^21,22^. In this study, RecurIndex, a multigene prognostic test, was used to assess the risk of LRR and DR in MBC. The results showed no statically significant association of RI-LR with MBC prognosis. However, RI-DR could effectively predict the DRFS, PFS and OS of MBC patients and identify those at low risk of recurrence to make them exempt from adjuvant chemotherapy. These findings suggested that RI-DR could be a potential prognostic tool for MBC.

RI-DR, a clinical-genomic model generated by clinical variables and genetic information, has a relatively high sensitivity and a relatively high negative predictive value. It is useful in the identification of low-risk breast cancer patients, particularly in those with Asian genetic backgrounds^23^. A previous study showed that RI-DR contributed to classifying Asian endocrine-responsive breast cancer patients with both negative and positive lymph nodes into the low- and high-risk groups based on 10-year DR^24^. In another study performed by Huang et al., it was also found a significant difference in 10-year DR-free intervals between low- and high-risk groups classified by RI-DR^19^. These studies all confirmed the utility of RI-DR in clinic.

Currently, National Comprehensive Cancer Network (NCCN) guidelines have proposed that multigene assays Oncotype Dx and MammaPrint can be used to evaluate the risk of recurrence in FBC according to certain cancer-gene expression patterns. In addition to providing information on recurrence prognosis, they also have predictive ability and can indicate who may benefit from additional chemotherapy^25^. With a high concordance rate with Oncotype Dx, RecurIndex possesses potential for helping clinicians make more informed decisions on adjuvant chemotherapy in Asian FBC patients^20,26^. Among the MBC patients without adjuvant chemotherapy in our study, the low-risk patients stratified by RI-DR tended to have better OS than the high-risk patients, and did not experience any recurrences or deaths, suggesting that the low-risk patients identified by RI-DR might be exempt from postoperative adjuvant chemotherapy. In a study performed by Giordano et al., 135 out of 156 MBC patients were identified no distant metastases, among whom 32 cases received chemotherapy, including 84% with adjuvant chemotherapy, 6% with neoadjuvant chemotherapy, and 9% with both. The results showed that 10-year OS rates of patients with lymph node-negative disease and those with lymph node-positive disease were 75% and 43%, respectively, indicating that the death risk of MBC patients with lymph node-positive disease did not decrease significantly after adjuvant chemotherapy^27^. There is another study showing that adjuvant chemotherapy may be skipped for stage I-IIA MBC patients^28^. Additionally, our results also revealed that 4 high-risk patients identified by RI-DR who developed recurrence or deaths might benefit from adjuvant chemotherapy, suggesting that adjuvant chemotherapy may be taken into consideration in MBC patients at high risk of recurrence or metastasis^29^. Hence, RI-DR can not only provide clinical outcome information before treatment, but also contributes to risk–benefit assessment of systematic adjuvant chemotherapy.

The major superiority of our study was that it first employed RecurIndex, a multigene prognostic test, to assess the prognosis of Chinese MBC patients and showed the clinical utility of RI-DR in MBC. Additionally, RI-DR can identify the patients at low risk of recurrence, probably leading to a reduction of adjuvant chemotherapy. It may be a necessary study whether MBC patients should receive postoperative adjuvant chemotherapy or not. However, there were also several limitations in our study. First, our study was a retrospective, single-center study with the small sample size, which may affect the statistical power and reliability of results. Second, we did not find statically significant association of RI-LR with MBC prognosis, which might be associated with the small sample size. In the future, more well-designed, large-scale studies should be implemented to further investigate the correlation between RI-LR and the prognosis in MBC.

## Conclusions

RI-DR can better predict the DRFS, PFS and OS of MBC patients and identify those at low risk of recurrence to make them exempt from adjuvant chemotherapy to the greatest extent, which may serve as a potential prognostic tool for MBC. However, the association of RI-LR with clinical outcomes in MBC still needs large-scale studies to further investigate.

## Methods

### Patients and data description

In this study, the clinicopathological data of 43 consecutive MBC patients undergoing surgeries in The Fourth Hospital of Hebei Medical University between 2009 and 2018 were analysed retrospectively. Their formalin-fixed and paraffin-embedded (FFPE) tumour samples were detected using RecurIndex test. Written consent forms were obtained from all the patients. The study was approved by the Institutional Review Board of The Fourth Hospital of Hebei Medical University (Approval No.: 20211372), and all the procedures performed were in accordance with relevant guidelines/regulations, as well as the 1964 Helsinki declaration and its later amendments.

The clinicopathological data of patients were collected by reviewing the electronic medical record, including age, LVI, tumour grade, tumour stage, Ki-67 expression, ER/PR and HER2 status, as well as presence or absence of adjuvant endocrine therapy, adjuvant chemotherapy and adjuvant radiotherapy.

### RecurIndex test

As an in vitro test, RecurIndex utilized real-time fluorescent quantitative nucleic-acid amplification technology to detect the ribonucleic acids (RNA) extracted from FFPE breast cancer samples, which was used for analysing gene-expression profiling of breast cancer. The primer pairs of genes included in RecurIndex test were complementary to the messenger RNA sequence of each target gene, and PanelStation was used for real-time fluorescent quantitative amplification. Based on gene-expression profiling of breast cancer and clinical factors, the risk scores of LRR and DR were calculated using analysis software.

A previous study has demonstrated that the best cut-off values of RecurIndex test for predicting LRR and DR are 8% and 4%, respectively^19^. According to the RI-LR and RI-DR generated from RecurIndex test, patients were separately divided into the low- and high-risk groups. Postoperatively, all the patients were followed up by further consultations and telephones. The follow-up deadline was February, 2020.

### Statistical analysis

The data in this study were managed using R software (version 4.0.1, The R Foundation). Normally distributed measurement data were expressed as mean ± standard deviation ($\stackrel{-}{x}$ ± s), *t*-test was used. Non-normal distribution data were presented as the median and interquartile [M (Q1, Q3)], the Mann–Whitney U rank sum test was performed. The ranked data were presented as number of cases and percentiles n (%), χ^2^ test or Fisher’s exact test was performed. The survival curves were drawn using Kaplan–Meier method and compared by Log-rank test. *P* < 0.05 was considered statistically significant.

### Ethics approval and consent to participate

The study was approved by the Institutional Review Board of The Fourth Hospital of Hebei Medical University (Approval No.: 20211372). All the patients included in this study had given informed consent, and all the procedures performed were in accordance with relevant guidelines/regulations, as well as the 1964 Helsinki declaration and its later amendments.

## Data Availability

The datasets used and/or analysed during the current study are available from the corresponding author on reasonable request.
